# Interaction between arbuscular mycorrhizal fungi and *Bacillus* spp. in soil enhancing growth of crop plants

**DOI:** 10.1186/s40694-019-0086-5

**Published:** 2019-11-28

**Authors:** Anuroopa Nanjundappa, Davis Joseph Bagyaraj, Anil Kumar Saxena, Murugan Kumar, Hillol Chakdar

**Affiliations:** 1 Centre for Natural Biological Resources and Community Development, 41 RBI Colony, Anand Nagar, Bangalore, 560024 India; 2 ICAR-National Bureau of Agriculturally Important Microorganisms, Mau, Uttar Pradesh 275103 India; 3 Government Science College, Nrupathunga Road, Bangalore, 560001 India

**Keywords:** AMF, *Bacillus*, Interaction, Plant nutrition

## Abstract

Soil microorganisms play an important role in enhancing soil fertility and plant health. Arbuscular mycorrhizal fungi and plant growth promoting rhizobacteria form a key component of the soil microbial population. Arbuscular mycorrhizal fungi form symbiotic association with most of the cultivated crop plants and they help plants in phosphorus nutrition and protecting them against biotic and abiotic stresses. Many species of *Bacillus* occurring in soil are also known to promote plant growth through phosphate solubilization, phytohormone production and protection against biotic and abiotic stresses. Synergistic interaction between AMF and *Bacillus* spp. in promoting plant growth compared to single inoculation with either of them has been reported. This is because of enhanced nutrient uptake, protection against plant pathogens and alleviation of abiotic stresses (water, salinity and heavy metal) through dual inoculation compared to inoculation with either AMF or *Bacillus* alone.

## Introduction

The soil is a life supporting system rich in microorganisms with many kinds of interactions that determines the growth and activities of plants. Microorganisms in soil providing nutrients to plants, protecting them from biotic and abiotic stresses, and boosting their growth and yield is well documented [12, 25]. Rhizosphere is the narrow zone of soil around plant roots very rich in microbial activity due to the presence of root exudates with nutrients, sloughed off root cells and mucilage released by the plant root. Rhizosphere harbours 10–50 times more bacteria and 5–10 times more fungi compared to soil away from the roots [60]. Interaction between microorganisms in the rhizosphere has profound effects on the growth, nutrition and health of plants in agro-ecosystems and in natural ecosystems [57]. Numerous studies have shown specific effects of plants on the abundance and composition of microorganisms in the rhizosphere. A recent study brought out that the plant growth strongly influences the fungal alpha diversity in the rhizosphere than bulk soil [70]. Interactions between microorganisms in the rhizosphere influence plant health directly by providing nutrition and/or indirectly by protecting against biotic and abiotic stresses. However, most of the studies on rhizosphere microorganisms focused on bacteria than fungi [49]. Of the different microorganisms colonizing the rhizosphere arbuscular mycorrhizal fungi (AMF) are unique because they are partly inside the root and partly outside the root, thus influencing other microorganisms in the soil and also plant growth. AMF are known for their evolutionary history. Plant and AMF association has evolved over at least 500 million years which has led many to suggest that AMF could have played a major role in the colonization of land by plants [59]. This association is one of the most ancient symbiotic relationship in the biological world. This hypothesis is also supported by recent molecular studies done on liverworts which are the most ancient plants [61].

AMF forming symbiotic association with higher plants facilitate uptake of diffusion-limited plant nutrients such as phosphorus, zinc, copper, etc. [13]. Phosphorus which is essential for plant growth has a defined role in plant metabolism such as cell division, development, photosynthesis, breakdown of sugar, nutrient transport within the plant, transfer of genetic characteristics from one generation to another and regulation of metabolic pathways [13]. Enhanced phosphorus uptake by mycorrhizal plants is well documented. Various mechanisms have been suggested for increased phosphorus uptake by mycorrhizal plants like external hyphae exploring greater volume of soil for phosphorus away from the root, effective phosphorus acquisition by external hyphae by production of phosphatases and smaller radii of absorptive system [13, 45]. Inoculation with efficient AMF enhancing nutrition, growth and yield of crop plants is well documented [23]. Several studies carried out under phosphorus deficient soils have brought out that AMF help in the phosphorus nutrition of crop plants to the extent of saving 50% P fertilizer application with no adverse effect on growth and yield of crops [40, 65]. These fungi also protect the plants against biotic and abiotic stresses [47, 53]. These fungi although not host specific exhibit host preference, thereby an efficient fungus for inoculating a particular host can be screened and selected [9, 13]. AMF are widely used in organic agriculture and plant nurseries to improve the growth of economically important species [17]. By mediating the nutritional flux between the plant and many microbes in the soil, AM symbiosis constitutes the backbone of the plant holobiont. Even though the importance of the AM symbiosis has been well recognized its circadian chronobiology remains almost completely unknown [42].

**Fig. 1:**
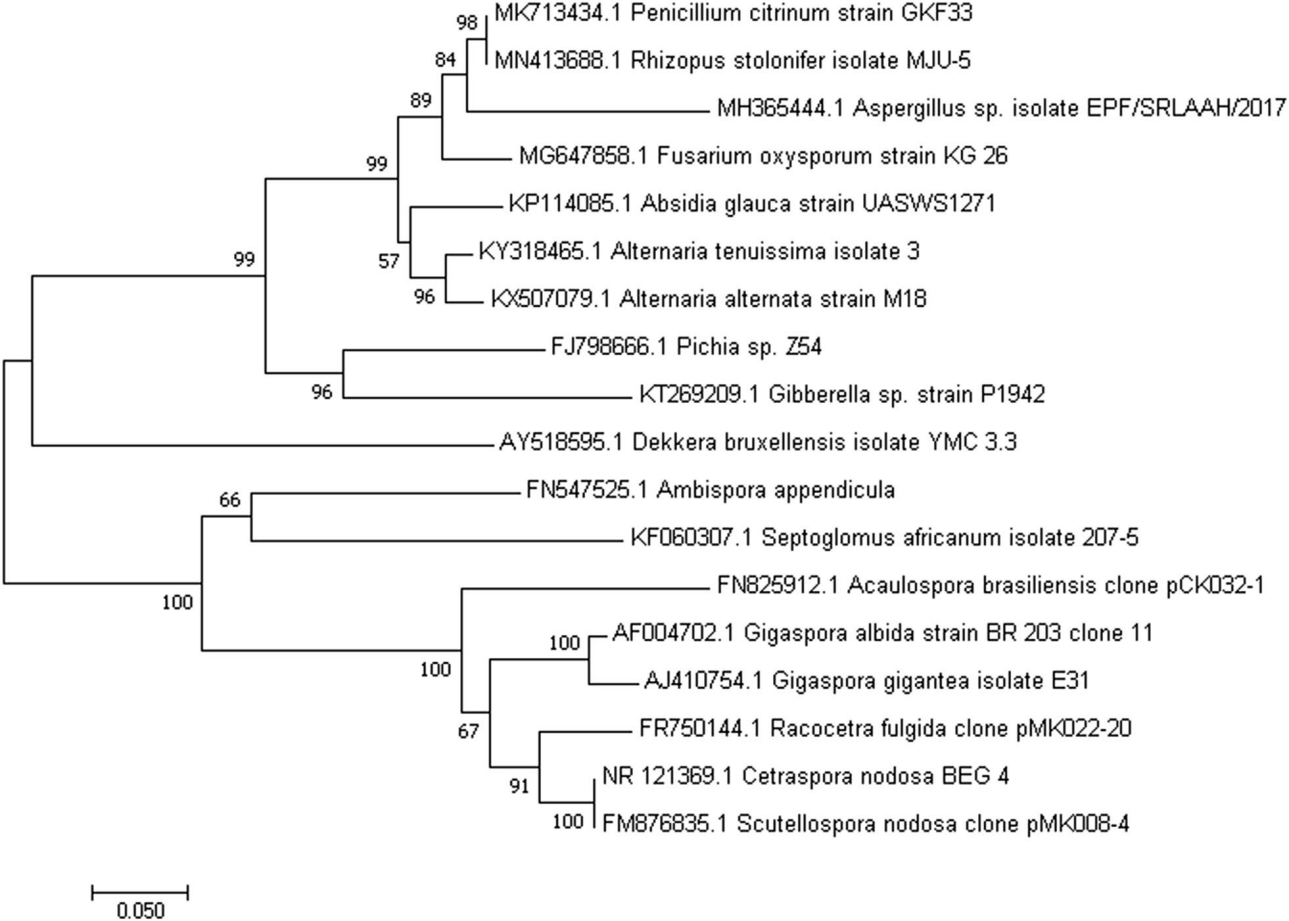
Internal Transcribed spacer (ITS) based phylogenetic analyses showing the relationship between selected free living fungi and AMF. The values at the nodes indicate bootstrap values. For phylogenetic analyses, ITS sequences were downloaded from NCBI, aligned using CLUSTAL-W option in MEGA 7 and phylogenetic tree was generated using Neighbour Joining method

AMF interact with wide range of microorganisms in the root and in the rhizosphere. AMF specifically harbor gene sets and metabolic machineries responsible for successful colonization in plant roots. Till date 321 species of AMF belonging to 36 genera have been described (http://www.amf-phylogeny.com). These unique species are phylogenetically quite diverse and have evolved differently from the free living fungi. Figure 1 shows a limited phylogenetic analysis of selected free living fungi and AMF, indicating their phylogenetic relationship. These fungi enhancing the number and activity of beneficial soil microorganisms with consequential beneficial effect on plant growth has been reported by earlier workers [2, 32]. In recent years there has been considerable interest on plant growth promoting rhizobacteria (PGPR), which improve plant growth by providing growth promoting substances and suppressing root pathogens [29, 55]. Synergistic interaction between AMF and PGPR benefitting the growth of plants compared to single inoculation with either of them has been reported by earlier workers [19, 24]. AMF and PGPR in soil and plant tissues mutually cooperate with each other in benefitting plant growth through increased nutrition, hyphal permeability in plant roots, bacterial survival and protection against biotic and abiotic stresses. Impressive molecular works have revealed a number of basic principles underlying plant–microbe interactions like (i) signals from microbes that are perceived by cognate plant immune receptors to initiate defense or symbiotic responses [39], (ii) microbial DNA and/or protein secretion systems that transport molecules into the host plant cell to modulate cell functions [18, 36]. Signaling between plants and microorganisms through transport signaling compounds is another important finding. Communication through signalling molecules, such as flavonoids, strigolactones and sesquiterpenes, is important for regulation of these interactions. Strigolactones released in low concentrations by rhizosphere microorganisms is known to facilitate colonization of plants by AMF. Among the PGPR, *Bacillus* is one of the important genus that exists in soil or as an endophyte and being a spore former with better saprophytic ability and competitiveness, it can survive in soil for long period of time under harsh environmental conditions. *Bacillus* spp. assist plants in its defense against pathogen attack and also enhance stress tolerance by inducing the expression of stress-response genes, phytohormones and stress-related metabolites [33]. The interactive effect of AMF with *Bacillus* spp. in soil and their potential to improve plant growth is discussed in this review.

### Synergistic interaction of AMF with *Bacillus* species in enhancing plant growth

Most of the *Bacillus* species directly stimulate plant growth either through enhancement in acquisition of nutrients or through stimulation of host plant’s defense mechanisms prior to infection or can associate with AMF and enhance plant growth [3]. Co-inoculation of AMF and PGPR has been proposed as an efficient method to increase plant growth by many workers. Several researchers have investigated the potential of AMF + *Bacillus* spp. association in enhancing the growth of plants (Table 1). Medina et al. [48] studied the effects of two *Bacillus* strains (*Bacillus pumilus* and *B. licheniformis*) on *Medicago sativa* plants with single or dual inoculation with three AMF and compared it with P-fertilization. The effectiveness of AMF species was determined by the bacterial strain associated for most of the plant parameters studied. The most efficient treatment was the dual *Glomus deserticola* +* B. pumilus* inoculation in terms of dry matter production. The different AMF had different effects on *Bacillus* spp. studied, indicating ecological compatibilities between microorganisms. Adriana et al. [1] investigated the interaction between three different AMF isolates (*Glomus constrictum* autochthonous (GcA); *G. constrictum* from collection (GcC); and commercial *Glomus intraradices* (Gi) and a *Bacillus megaterium* (Bm) strain isolated from Mediterranean calcareous soil and their effect on *Lactuca sativa* plant growth. Inoculation with the consortium (GcA + Gi + Bm) increased plant growth but decreased when Bm was in combination with GcC. Plants inoculated with GcC + Bm had highest glucose-6-phosphate dehydrogenase (G6PDH) and the lowest glutamine synthetase (GS) enzymatic activities, whereas Gi + Bm inoculated leaves showed the highest GS activity and it is well known that these enzymatic activities are related to plant growth and performance.

**Table 1:** Interaction between AMF and *Bacillus* spp. promoting plant growth

AMF	*Bacillus* spp.	Plant	References
*Glomus deserticola* and two other AMF	*B. pumilus* and *B. licheniformis*	*Medicago sativa*	Medina et al. [48]
*G. constrictum G. intraradices*	*B. megaterium*	*Lactuca sativa*	Adriana et al. [1]
*G. fasciculatum*	*B. subtilis*	*Tagetes erecta*	Flores et al. [27]
*G. mosseae*	*B. subtilis*	*Artemisia annua*	Awasthi et al. [10]
*G. aggregatum, G. fasciculatum, G. intraradices* and *G. mosseae*	*B. subtilis*	*Pelargonium graveolens*	Alam et al. [5]
*G. intraradices*	*B. polymyxa *+other PGPR	*Stevia rebaudiana*	Vafadar et al. [66]
*G. mosseae*	*B. subtilis* +other PGPR	*Cucumis sativus*	Rabab [58]
*Funneliformis mosseae*	*B. sonorensis*	*Capsicum annuum*	Thilagar et al. [65]
*Acaulospora laevis* and *Claridioglomus etinucatum*	*B.licheniformis*	*Withania somnifera*	Anuroopa and Bagyaraj [6]
*Funneliformis mosseae*	*B. sonorensis*	*Solanum lycopersicum* and *Capsicum annuum*	Desai et al. [22]

Interaction between *Glomus fasciculatum* and *Bacillus subtilis* was studied by Flores et al. [27] on marigold for flower yield and quality. The plants were inoculated with *Glomus* and/or *Bacillus* at sowing and transplanting time. The dual inoculated plants produced nearly 20% more inflorescence than uninoculated plants. Flowers in the inoculated treatment did not differ in size, however they had significantly higher fresh weight than control. The AMF improved xanthophyll content where as the bacterium enhanced flower clarity and yellow color. Awasthi et al. [10] reported that dual inoculation with *Glomus mosseae* and *Bacillus subtilis* increased the artemisinin content in the medicinal plant *Artemisia annua*, while individual inoculation with *Glomus mosseae* or *Bacillus subtilis* was not effective in increasing artemisinin content. Inoculation of *Bacillus subtilis* with four different AMF, *Glomus aggregatum, Glomus fasciculatum, Glomus intraradices* and *Glomus mosseae*, alone and in combinations were evaluated for the productivity of geranium by Alam et al. [5]. Plants inoculated with the consortium of *B. subtilis* + *G. mosseae* significantly increased the herb yield and the total oil yield over untreated control which was validated by field experiment.

Vafadar et al. [66] studied the effect of AMF *Glomus intraradices*, and PGPR *Bacillus polymyxa, Pseudomonas putida* and *Azotobacter chroococcum* on *Stevia rebaudiana*. The results showed increased effects due to dual compatible mixtures of inoculants resulting from their strong synergistic relationship among themselves. All growth parameters including stevioside content recorded were significantly higher in plants inoculated with *G. intraradices *+ *B. polymyxa*. Similarly, Anuroopa and Bagyaraj [6] investigated the effect of individual as well as microbial consortia of *Acaulospora laevis*, *Claridioglomus etinucatum* and *Bacillus licheniformis* on the growth of *Withania somnifera*. Plant growth, dry biomass, plant nitrogen and phosphorus, withanolide concentration, mycorrhizal spore count and root colonization were found to be maximum in plants inoculated with *A. laevis* +* B. licheniformis* when compared with individual inoculated treatments and uninoculated plants. Rabab [58] conducted a field experiment to study the interaction of *Bacillus subtilis* and *Trichoderma harzianum* with AMF *Glomus mosseae* on growth parameters of *Cucumis sativus.* The study showed that the consortium increased the number of mycorrhizal spores, root colonization and infection index of AMF, and increased the growth and yield of cucumber plant. Thilagar et al. [65] screened and selected the best AMF *Funneliformis mosseae* and PGPR *Bacillus sonorensis* for inoculating chilly and later found that dual inoculation is the best for inoculating chilly through pot culture studies. Further microplot experiment conducted with varying levels of chemical fertilizers in order to reduce the recommended level of fertilizers for chilly cultivation brought out that 50% of the recommended NPK fertilizers can be reduced with no adverse effect on growth, nutrition and yield of chilly with dual inoculation. Large scale field trial conducted at farmer’s field validated the microplot results. Desai et al. [22] inoculated *Bacillus sonorensis *and *Funneliformis mosseae* to the planting medium in pro trays to raise tomato and capsicum seedlings in a polyhouse. The results revealed that the inoculation with consortium is beneficial for raising healthy, vigorously growing tomato and capsicum seedlings in pro trays under polyhouse condition.

### Interaction of AMF with *Bacillus* spp. in protecting plants against pathogens

Priming of plant immune system in response to biological agents is a common practice, which enables plants with augmented capability to defend pathogen attack. Symbiotic AMF and PGPR are known to induce systemic resistance to soil-borne pathogens. The presence of these microorganisms in the soil/rhizosphere or intentional introduction of these microorganisms to the soil helps to improve plant’s general health and its ability to carry out its physiological functions to the best of its potential [14]. One of the major factors regulating root microbiome structure is the interactions between mycorrhizal fungi, soil bacteria and the plant, which play a crucial role in shaping the microbiome community. Host plant genotype strongly influences the extent to which AMF and PGPR colonize the host roots through the production of root exudates that attract specific microorganisms to the rhizosphere. Certain chemicals like strigolactones and benzoxazinoids produced by plants induce positive chemotaxis and help to recruit specific AMF [4] and PGPR respectively near the root zone [54]. Several researchers have studied the possibility of combined inoculation and reported that plants show preferences for this kind of association. *Bacillus* species being an important soil genus has been investigated in such association studies with AMF (Table 2).

**Table 2:** Interaction between AMF and *Bacillus* spp. in protecting plants against plant pathogens

AMF	*Bacillus* spp.	Pathogen	Plant	References
*Glomus mosseae* or *G. manihotis*	*Bacillus* sp.	*Meloidogyne incognita*	*Carica papaya*	Jaizme-vega et al. [37]
*Glomus* spp.	*Bacillus* sp	*Verticillium dahliae*	*Fragaria ananassa*	Tahmatsidou et al. [64]
AMF	*B. amyloliquefaciens* +other PGPR	*Fusarium oxysporum* f. sp. *radicis*-*lycopersici*	*Solanum lycopersicum*	Yusran et al. [74]
*G. aggregatum*	*B. coagulans*	*Meloidogyne incognita*	*Solanum lycopersicum*	Serfoji et al. [62]

Jaizme-Vega et al. [37] studied the combined inoculation of two AMF species *Glomus mosseae* or *G. manihotis* and a *Bacillus* spp. consortium in reducing nematode infestation and damage in papaya. Plants were harvested 160 days after nematode inoculation. Dual inoculation with AMF + *Bacillus* spp. significantly reduced the *Meloidogyne* infestation and resulted in enhanced plant growth. Biological control of wilt caused by *Verticillium dahliae* in strawberry based on single and dual inoculation with a commercial AMF inoculant containing *Glomus* spp. and a commercial PGPR inoculant containing a *Bacillus* sp., was evaluated by Tahmatsidou et al. [64] in the field. Dual inoculation did not give greater protection than single inoculation bringing out that the commercial inoculants used by them were not of good quality. Yusran et al. [74] tested the efficacy of two commercial bacterial strains *Pseudomonas* sp. and *Bacillus amyloliquefaciens* in improving mycorrhization, nutrient status and plant growth of tomato affected by *Fusarium oxysporum* f. sp. *radicis*-*lycopersici*. Combined inoculation with the bacterial strains and AMF increased the observed effects on dry matter and shoot nutrient concentrations. A glass house experiment was conducted by Serfoji et al. [62] to check the effectiveness of *Glomus aggregatum* and *Bacillus coagulans* along with vermicompost for the management of *Meloidogyne incognita* on tomato cultivar Pusa Ruby. The AMF alone and the consortium resulted in maximum growth, biomass and nutrients in tomato with decreased root- knot nematode population and root- knot index. Application of vermicompost along with *G. aggregatum* and *B. coagulans* further increased plant growth and mycorrhizal colonization but decreased root- knot nematode reproduction rate, number of galls and egg mass.

### Interaction of AMF with *Bacillus* spp. in alleviating abiotic stress

Plant growth is benefitted by addition of AMF and PGPR which not only helps to increase germination rate, root growth and shoot and root weight, grain yield, chlorophyll content, but also induce tolerance to drought, salt stress and delay senescence. There are publications reporting that AMF interact with *Bacillus* spp. to increase plant growth under stress (Table 3). The effect of dual inoculation with AMF *Glomus mosseae* or *Glomus intraradices* and PGPR, *Bacillus* sp. was investigated by Vivas et al. [69] on the development and physiology of lettuce. Plants were assessed for growth, mineral nutrition and gas-exchange in response to microbial inoculation after polyethylene glycol (PEG) induced drought stress. In plants, inoculated with AMF + *Bacillus* sp. there was increase in fungal development and succinate dehydrogenase (SDH) and alkaline phosphatase (ALP) activities, and also plant growth. *Bacillus* sp. inoculation improved all the plant and fungal parameters to the same level as in non-stressed plants. The results clearly brought out the benefit of co-inoculation with AMF + *Bacillus* sp. in alleviating water stress. Marulanda et al. [46] evaluated the interactions between *Bacillus thuringiensis*, a drought-adapted bacterium, and two isolates of *Glomus intraradices* (an indigenous drought-tolerant and a non indigenous drought-sensitive), on *Retama sphaerocarpa,* a drought-adapted legume. Maximum root development, nodule numbers, mycorrhizal colonization, plant growth and water uptake were observed in plants co-inoculated with *Bacillus thuringiensis* plus the indigenous drought tolerant isolate of *Glomus intraradices.*

**Table 3:** Interaction between AMF and *Bacillus* spp. to alleviate abiotic stress

AMF	*Bacillus* spp.	Abiotic stress	Plant	References
*Glomus mosseae* and *G. intraradices*	*Bacillus* sp.	Drought	*Lactuca sativa*	Vivas et al. [69]
*G. intraradices*	*B. thuringiensis*	Drought	*Retama sphaerocarpa*	Marulanda et al. [46]
AMF	*B.cereus* + *Candida parapsilosis*	Heavy metal	*Trifolium repens*	Azcón et al. [11]
*G. etunicatum*	*B. subtilis*	Surfactant		Xiao et al. [71]
*Rhizophagus intraradices*	*B. thuringiensis *+* B. megaterium *+* Pseudomonas putida*	Drought	*Trifolium repens*	Ortiz et al. [56]
Consortium of AMF	*B. thuringiensis*	Drought	*Zea mays*	Armada et al. [7]
Single autochthonous AMF	*B. thuringiensis*	Drought	*Lavandula dentata*	Armada et al. [8]
*Claroideoglomus etunicatum* + *Rhizophagus intraradices* + *Funneliformis mosseae*	*B. subtilis*	Salinity	*Acacia gerrardii*	Hashem et al. [33]

Armada et al. [7] investigated the effectiveness of a drought-adapted AMF and *Bacillus thuringiensis* consortium to improve plant growth and physiology in maize under drought stress. Several physiological parameters including the expression of plant aquaporin genes were measured. Inoculation resulted in increased plant nutrition, plant drought tolerance including regulation of plant aquaporins with several putative physiological functions. A similar work carried out by Ortiz et al. [56] using autochthonous AMF and *Bacillus thuringiensis* on *Trifolium repens* also brought out that inoculation enhanced drought tolerance in plants compensating for the detrimental effect of water limitations. Armada et al. [8] evaluated the response of *Lavandula dentata* under drought conditions to inoculation with an autochthonous AMF and native *Bacillus thuringiensis* (endophytic bacterium) singly and together. Inoculation with the consortium increased plant growth and nutrition and increased drought tolerance and antioxidant activities such as superoxide dismutase, catalase and ascorbate peroxidase. There was increased mycorrhizal development, indole acetic acid and 1-aminocyclopropane-1-carboxylate (ACC) deaminase production and phosphate solubilization indicating its capacity to improve plant growth under stress conditions. The autochthonous AMF species and particularly their combination with *B. thuringiensis* demonstrated the potential for protecting plants against drought and helping plants to thrive in semiarid ecosystems.

Hashem et al. [33] conducted a greenhouse experiment to examine synergistic impact of the AMF, *Claroideoglomus etunicatum*; *Rhizophagus intraradices* and *Funneliformis mosseae*; and PGPR, *Bacillus subtilis* to induce acquired systemic resistance in Talh tree (*Acacia gerrardii*) against adverse impact of salt stress. Compared to the control, the *Bacillus subtilis* treatment significantly enhanced root colonization intensity by AMF, in both presence and absence of salt. They also found positive synergistic interaction between *B. subtilis* and AMF in terms of increase in total lipids, phenols, and fiber content. The *B. subtilis* + AMF inoculated plants showed increased content of osmoprotectants such as glycine, betaine and proline. The application of these microbial inoculants to the tree turned out to be beneficial in reducing the deleterious effect of salt on plant metabolism, probably by modulating the osmoregulatory system and antioxidant enzyme system. The effect of different AMF (*G. fasciculatum*, *G. mosseae*, *G. aggregatum*) and the PGPR *B. pumilus* on growth *Ocimum basilicum* grown under 40 ppm of sodium fluoride stress was investigated by Yadav [72]. Dual inoculation with AMF + *B. pumilus* showed a remarkable increase in plant height, leaf fresh weight, leaf dry weight and total fresh biomass. Consortium of *G. mosseae* + *B. pumilus* resulted in 24% increase in leaf fresh weight and also increased the fluoride tolerance level of the herb.

The biocompatibility between AMF *Glomus etunicatum* and a biosurfactant-producing bacterial strain *Bacillus subtilis* was investigated by Xiao et al. [71]. The effect of *B.* *subtilis* on the mycoremediation of soils artificially contaminated with different levels of phenanthrene was investigated in pot experiments. Mycorrhizal or *B. subtilis* inoculation improved the tolerance to stress of phenanthrene and increased the plant biomass. Biosurfactant secreted by *B. subtilis* considerably enhanced the solubility of phenanthrene, favouring its enrichment in rhizosphere soil and plant roots. The co-inoculation of *G.* *etunicatum* and *B. subtilis* significantly decreased the residual concentrations of phenanthrene in soil, and resulted in higher soil enzyme activities of catalase and polyphenol oxidase. Therefore, inoculation of biosurfactant-producing strain of *Bacillus* + AMF *G. etunicatum* could be a potential biotechnological approach for the remediation of soil polluted with polycyclic aromatic hydrocarbons. Azcón et al. [11] investigated the development of *Trifolium repens* growing in a heavy metal contaminated soil inoculated with different microorganisms. The plant growth was increased by selected native microorganisms, *Bacillus cereus*, *Candida parapsilosis* or AMF, used either as single or dual inoculants. The dual inoculation with AMF + *B. cereus* increased plant biomass compared to other treatments. The AMF colonization and nodulation was negligent in plants growing in this natural, polluted soil which was compensated by AMF + *B. cereus* inoculation. The metal bioaccumulation abilities of the inoculated microorganisms and particularly the microbial effect on decreasing metal concentrations in shoot biomass seem to be involved in such effects. Inoculation with AMF + *B. cereus* showed a bioremediation potential and helped plants to develop in the contaminated soil. Thus, they could be used as a biotechnological tool to improve plant development in heavy metal contaminated environments. From the studies conducted so far on soil AMF + *Bacillus* spp. it can be concluded that the information available is scanty, suggesting more investigations are needed in this area.

### Interaction of AMF with endosymbiotic bacteria

AMF host intracellular bacteria that can colonize the surface of spores and hyphae which affect spore germination, hyphal growth, and root colonization [35, 43]. Morphological and genetic approaches of genes related to metabolism, cell colonization events and nitrogen fixation suggests a potential role in the nutritional exchanges between endobacteria, fungi and plants [51]. AMF also benefit from the production of bacterial metabolites such as organic acids, volatile compounds (ethylene), and non-volatile compounds [34]. Some endobacteria are obligate biotrophs, not able to grow without AMF [38]. Interaction of AMF, bacteria and plants brings another level of complexity to diversity and function of the mycorrhizal symbiosis and can be considered as tripartite associations resulting in a consortium that promotes plant growth [16]. In addition, the diversity of these associated bacteria has not been explored. Lumini et al. [43] established that the presence of endosymbiotic bacteria strongly improves the presymbiotic growth by comparing lines of *Gigaspora margarita* harbouring endosymbiotic *Candidatus* Glomeribacter gigasporarum with lines that have been cured. Cruz et al. [21] isolated three bacterial strains from spores of *Gigaspora margarita*. The bacteria were identified by morphological methods and on the basis of ribosomal gene sequences as *Bacillus* sp. (KTCIGM01), *Bacillus thuringiensis* (KTCIGM02), and *Paenibacillus rhizospherae* (KTCIGM03). The probable endobacteria suppressed soil-borne plant pathogens, promoted hyphal growth, and stimulated nutrient biodynamics, as reflected by phosphorus solubilization and nitrogenase activity measurements. However, the potential roles and infection mechanisms of these bacteria, in particular the endobacteria, are still poorly understood. Some of the bacteria associated with spores and hyphae of AMF are known to enhance colonization and function of AMF, which are referred to as mycorrhiza helper bacteria (MHB). *Bacillus coagulans* isolated from the hyphae of *Rhizophagus fasciculatus* was the first report on MHB occurrence in AMF [44] followed by several other reports [16, 28, 73]. Therefore, it is possible that MHB and AMF can positively interact to promote a sustainable nutrient supply to plants [28]. Some of these MHB can be endosymbionts. Thus the interaction between plant, AMF with endosymbionts and rhizosphere bacteria is complex and needs more investigation.

### Molecular interactions between plants, AMF and soil bacteria

Interaction of AMF with plants and other rhizospheric microbes is complex and very intricate [20]. An exchange of molecular signals among the participants ensures a successful interaction. Host plants can release specific signal molecules (e.g. Strigolactone) which when perceived by the mycorrhizal fungi results in extensive hyphal branching leading to increase probability of root-fungal contact. Similarly, mycorrhizal fungi secrete certain signal molecules known as “myc factors” which can activate morphological and physiological changes in plants through induction of “sym pathway” [26, 30]. It has been deduced that seven proteins viz. SYMRK (symbiosis receptor kinase), DMI2 (DOES NOT MAKE INFECTIONS2); cation channels (CASTOR and POLLUX); nuclear porins (NUP85 and NUP133) are necessary for the induction of Ca^2+^ spiking and CCAMK/DMI3 (calcium/calmodulin-dependent protein kinase) are required for transducing the calcium signals. CCAMKDMI3 interacts with CYCLOPS (IPD3) and is required for mycorrhizal colonization [41, 50]. It has been reported that more than 10,000 genes are involved in AMF-plant symbiosis. As the physiology of plants change due to AMF colonization, the composition of the root exudates also changes and affects the microbial communities in the rhizosphere. Still not much is actually known about the molecular cross-talk between *Bacillus* and AMF. It has been reported that the receptors for the signal molecules produced by beneficial bacteria and AMF share significant homology and even in some cases same receptors can perceive both the signals. Exopolysaccharides produced by beneficial bacteria have been attributed as an important factor for establishing association with AMF [15]. Certain exoribonuclease related genes have also been described in beneficial endosymbiotic bacteria which are required for developing association with *Gigaspora margarita* [67]. AMF hyphae have been reported to release organic compounds which can act as source of energy for rhizospheric microbes [52]. Guether et al. [31] used Affymetrix GeneChip to understand the transcriptional changes in *Lotus japonicas* upon colonization with *Gigaspora margarita* and 558 genes were found differentially expressed. SCARECROW family of transporters, phosphate transporters, ammonium transporters, potassium transporters were found to be significantly upregulated. Expression profiling using RNASeq revealed that inoculation of *Rhizoglomus irregulare* resulted in modulation of 726 genes in Sunflower roots and especially genes involved in membrane transport and cell wall shaping were significantly upregulated [68]. Although considerable progress has been made to understand the molecular signaling using Next Generation Sequencing (NGS) technologies, CRISPR based gene knock in/out, gene transfer/replacement technologies can be used as powerful tools to dissect the plant–microbe interactions to the next level [63]. CRISPR tools along with other gene editing technologies like TALENs (Transcriptor Activator Like Effectors Nucleases) can be very helpful to manipulate key regulatory genes involved in plant-AMF-bacteria interactions leading to improved AMF inoculants with better efficiency.

A circadian rhythm is a natural, internal process that regulates the various biological function which repeats roughly every 24 h. These 24-h rhythms are driven by a circadian clock, and they have been widely observed in plants and also in microbes. Circadian clocks are nearly ubiquitous timing mechanisms that can orchestrate rhythmic behavior and gene expression in a wide range of organisms. The arbuscular mycorrhizal (AM) symbiosis, formed by plant roots and fungi, is one of the oldest and most widespread associations between organisms. By mediating the nutritional flux between the plant and the many microbes in the soil, AM symbiosis constitutes the backbone of the plant holobiont. Even though the importance of the AM symbiosis has been well recognized its circadian chronobiology remains almost completely unknown [42].

### Future perspective

The current day emphasis is on sustainable agriculture. It implies use of natural resources like beneficial soil microorganisms for producing food and enhancing quality of the environment. AMF being a part of microorganisms occurring in the rhizosphere attempts to integrate them with other beneficial soil organisms should be investigated for holistic plant and soil health management. There is a paucity of well controlled studies on the use of microbial interactions to improve productivity in cropping systems. Considerable research has been carried out on inoculating plants with AMF and *Bacillus* spp. separately and showing their beneficial effect on plant growth. Using AMF and *Bacillus* spp. together as a consortium in enhancing plant growth and productivity is scanty and needs more investigations. The plant-AMF-*Bacillus* interaction being complex more molecular studies on the cross-talk between the three biological systems are needed to understand their intricate interaction.

## Conclusion

AMF and *Bacillus spp.* in soil can act synergistically with each other and promote plant growth in much bigger way, compared to inoculation singly with either of them. This is due to complementary impact on soil nutrient solubilization and uptake. Combined inoculation also helps in alleviating plants against plant pathogens and abiotic stresses like drought, salinity, heavy metal toxicity etc. Dual inoculation with AMF and *Bacillus* spp. under field conditions brought out that 50% of recommended NPK fertilizers can be reduced with no adverse effect on growth, nutrition and yield of crop plants. Studies on dual inoculation with AMF plus *Bacillus* spp. under field conditions are limited suggesting that more investigations are needed in this area.

## Data Availability

Not applicable.

## Abbreviations

AMF: arbuscular mycorrhizal fungi
PGPR: plant growth promoting rhizobacteria
P: phosphorus
spp.: species
GcA: *Glomus constrictum* autochthonous
GcC: *G. constrictum* from collection
Gi: *Glomus intraradices*
Bm: *Bacillus megaterium*
G6PDH: glucose-6-phosphate dehydrogenase
GS: glutamine synthetase
WUE: water use efficiency
ACC: 1-aminocyclopropane-1-carboxylate
